# Editorial: Moving towards a sustainable and greener dialysis

**DOI:** 10.3389/fmed.2025.1754961

**Published:** 2025-12-16

**Authors:** Sabrina Haroon, Andrew Davenport

**Affiliations:** 1 Division of Nephrology, National University Hospital, Singapore, Singapore; 2 Division of Medicine, University College London (UCL) Centre for Kidney & Bladder Health, Royal Free Hospital, University College London, London, United Kingdom

**Keywords:** carbon emissions, green dialysis, hemodialysis, peritoneal dialysis, renal replacement therapy, sustainability

The global prevalence of chronic kidney disease is increasing with a corresponding increase in end-stage kidney disease (ESKD) requiring dialysis. Current estimates indicate that approximately four million patients depend on dialysis as a life-sustaining treatment. Additionally, kidney replacement therapy is frequently employed in acute settings, often using continuous renal replacement therapy or slow low-efficiency dialysis. The environmental footprint of dialysis is substantial, primarily attributable to pharmaceuticals, but also to carbon emissions generated by single-use consumables, as well as the consumption of water and electricity.

The increasing global demand for dialysis, compounded by a shift toward offering newer, more complex treatments, such as hemodiafiltration and automated peritoneal dialysis (APD), exacerbates these environmental concerns. These modalities necessitate greater consumption of water, electricity, and consumables, thereby amplifying carbon emissions. Striking a balance between optimal clinical outcomes and environmental sustainability remains a critical and delicate balance. It is also pertinent to note that emerging technologies, such as sorbent-based systems, which are purported to be environmentally advantageous by reducing water usage, have not necessarily been evaluated for their environmental impact (1).

Prior research in dialysis units has highlighted the significant contribution of single-use consumables to dialysis units' carbon footprint; however, most studies lack direct measurements of emissions from manufacturing vendors (2–5). Instead, studies have generally used either a bottom-up approach, estimating greenhouse gas (GHG) emissions from material production, transport, dialysis use, and waste disposal, or a top-down approach based on item costs. As such, most current estimates have simply relied on financial data or computations based on previously published estimates, which may be inaccurate. Therefore, strategies to reduce carbon emissions from the standpoint of consumable manufacturers depend heavily on the reporting practices of dialysis manufacturers and regulatory policies at the national level. Although regulatory and policy efforts are underway and have been adopted more aggressively in recent years, reducing carbon emissions associated with manufacturing processes will take time, necessitating supplementary strategies.

**Figure 1:**
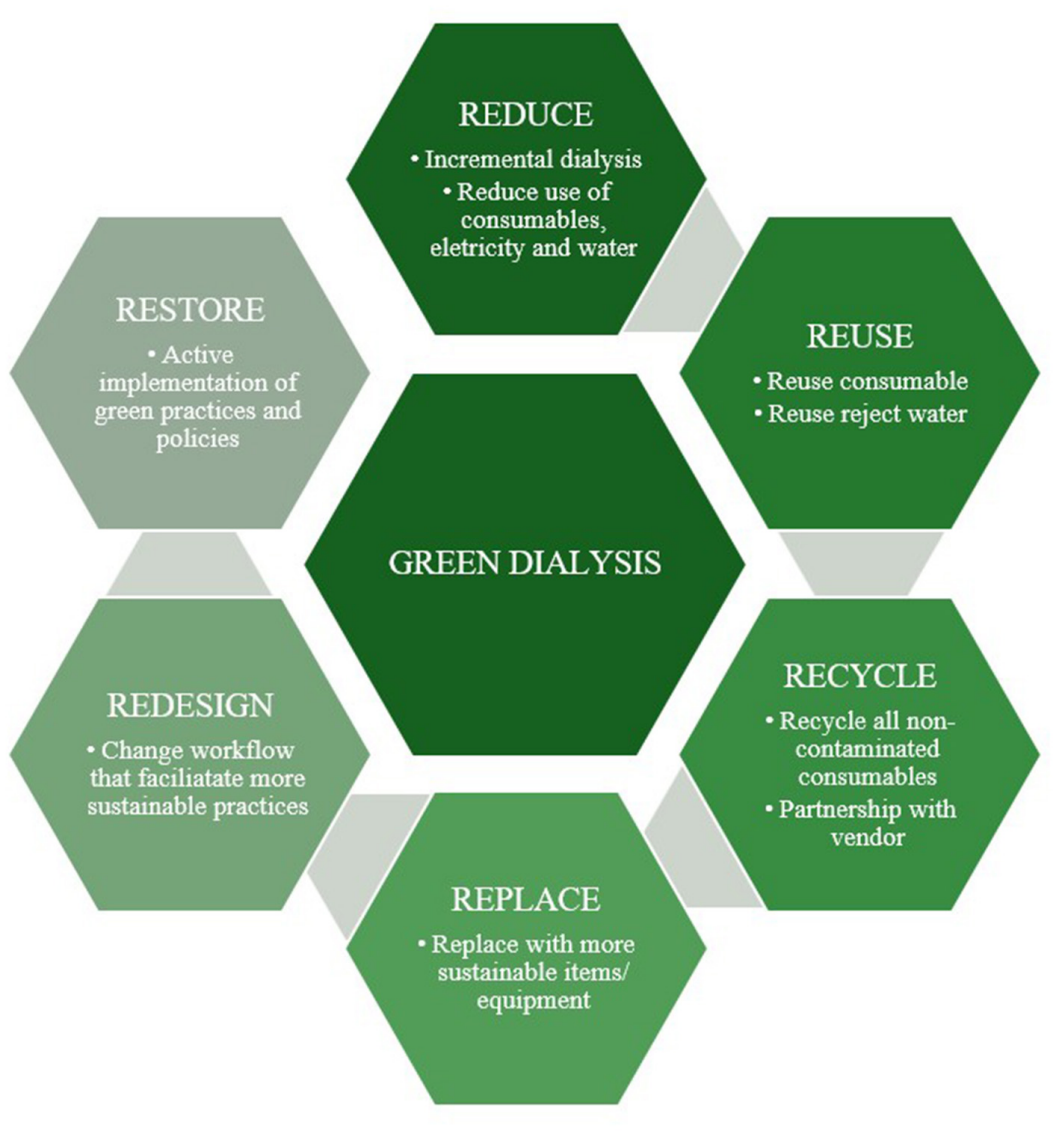
“Green dialysis” concepts: reduce, reuse, recycle, replace, redesign, and restore.

Fundamental principles of sustainability, notably reduce, reuse, recycle, and redesign, is central to addressing this issue (Figure 1). As more and more ESKD patients start dialysis with some residual kidney function, several studies have shown that many patients can safely start with an incremental approach, such as less frequent hemodialysis sessions or fewer peritoneal dialysis (PD) exchanges or treatment days. Thus, it requires fewer consumables and generates less waste, thereby reducing GHG emissions and health care costs. Nardelli et al., in their review, advocate a personalized approach to dialysis, emphasizing the importance of tailoring treatment to individual patient needs, thereby avoiding unnecessary treatment in those with significant residual kidney function. Personalized dialysis extends beyond simply minimizing carbon emissions, as additional work is required to tailor dialysis prescriptions, monitor changes in residual kidney function, and adjust prescriptions. Although this additional work may increase some health care costs, it offers benefits for workflow in the dialysis unit. With incremental patients dialyzing for fewer sessions or shorter duration, this potentially allows other patients to dialyze more, as needed, compared to a strict thrice-weekly 4-hour schedule. These principles have also been incorporated into hemodialysis practices, transcending conventional protocols (6). Similarly, in the intensive care setting, clinical practice has turned away from high-volume continuous hemofiltration/hemodiafiltration to more modest 20–25 mL/kg/h exchanges, which reduces not only the number of sterile bags of dialysate used per day, but also reduces the need for replacement of phosphate and magnesium and reduces nutrient and antibiotic losses. Molano-Triviño et al. expand on introducing the concept of tailoring continuous renal replacement dosing to the metabolic requirements of the individual patient.

In addition to incremental dialysis practices, several studies have reported that dialysis efficiency can be retained using slower dialysate flow rates. Although the dialysate water flow is counter-current to the blood flow, as the dialysate enters and leaves the dialyzer below the header, the dialysate flow may not permeate equally between all the capillary fibers in the tight bundle. Studies from some years ago reported more efficient sessional urea clearance at dialysate flow rates of 700–800 mL/min, due to better matching of dialysate-to-blood flow. However, following advances in dialyzer casing design and the baffle, which directs the dialysate water flow, this has now permitted efficient treatments with slower dialysate flows. Several dialysis machines are now fitted with autoflow devices, matching dialysate flow to blood flow, using 1:1.5 for hemodialysis and 1:1.2 for post-dilutional hemodiafiltration (7). Castillo et al. now report that using dialysate flows below the standard 500 mL/min not only reduces the clearance of small molecules but also that of middle-sized molecules. Depending on the water plant, around 50% of potable domestic water is lost in producing high-quality dialysis water, so a reduction in dialysate flow of 100 mL/min during a 4-h session could save 48 L of potable water. Other GHG savings can be achieved by switching from single-use acid concentrate plastic containers to either on-site production of acid concentrate or a central acid delivery system, and similarly by using smaller bicarbonate pouches (8).

Turning to re-use, historically, all dialyzers were reused. However, in many countries, re-use was discontinued as dialyzer costs fell, and concerns arose about reduced dialyzer clearances and patient safety (9). However, dialyzer re-use worldwide, particularly in low and middle-income countries, continues. Most studies have reported on urea clearance, but Prapunwatana et al. now report on middle-sized molecule clearances in a small series, suggesting that, whereas reuse led to a small reduction in β2 microglobulin clearance, there was a greater reduction in the clearance of larger molecules, with less sessional albumin loss.

This topic highlights multiple strategies to enhance the sustainability of dialysis practices, emphasizing approaches that can be implemented independently of manufacturer-led initiatives. While patient outcomes remain essential, foundational concepts such as minimizing carbon footprints, promoting recycling, and establishing environmentally-friendly clinical environments cannot be neglected. The aim is to inform practice with solutions that are both feasible and effective, fostering the development of sustainable dialysis modalities whilst optimizing patient outcomes.
